# A new approach to assessment for young children referred by education professionals for socio‐emotional, behavioural, and cognitive difficulties

**DOI:** 10.1002/jcv2.70138

**Published:** 2026-06-03

**Authors:** Amy L. Paine, Steve Eaton, Aikaterini Bekiropoulou, Christopher Hobson, Cerith Waters, Anita Thapar, Katherine H. Shelton, Kate Langley, Catherine R. G. Jones, Dale F. Hay, Susan R. Leekam, Elisabeth von dem Hagen, The Neurodevelopment Assessment Unit Team, Stephanie H. M. van Goozen

**Affiliations:** ^1^ School of Psychology Cardiff University Centre for Human Developmental Science Cardiff University Cardiff UK; ^2^ Wolfson Centre for Young People's Mental Health Cardiff University Cardiff UK; ^3^ Centre for Neuropsychiatric Genetics and Genomics Cardiff University Cardiff UK

**Keywords:** cognitive systems, latent profile analysis, mental health, neurodevelopment, systems for social processes, transdiagnostic

## Abstract

**Background:**

Young children with emerging mental health problems and neurodevelopmental differences often do not receive the support they need early in life, and if they do receive support, it may not be appropriately targeted towards their individual needs. The Neurodevelopment Assessment Unit (NDAU) was established to meet this need, offering an innovative method for evaluating young children through developmental profiling guided by the NIMH Research Domain Criteria (RDoC).

**Methods:**

Children (4–7 years) identified as experiencing social, emotional, and/or cognitive difficulties by education practitioners were referred to the NDAU to complete a comprehensive battery of assessments. We present an overview of the first cohort of children (*N* = 486) who were referred between September 2017 and January 2023. We first provide an overview of children's performance on constructs within the Cognitive Systems and Systems for Social Processes domains of the RDoC. As an exploratory analysis, we used latent profile analysis to identify subgroups of children with similar patterns of performance on assessments within these domains.

**Results:**

Children demonstrated most difficulties with recognition of negative emotions (81.6% below average in at least one negative emotion), understanding mental states (51.4% below average), and sustained attention (47.6% below average). Next, we identified three distinct subgroups of children, each defined by unique patterns of performance across Cognitive Systems and Social Processing domains.

**Conclusion:**

These findings highlight the NDAU as a promising model for dimensional assessment and underscore the importance of investigating variability and heterogeneity in the neuropsychological profiles of young children with emerging difficulties.

## Key Points

**What's known?**

- Many children with emerging mental health or neurodevelopmental features are often overlooked by support services.
- Characterising children's neuropsychological profiles can improve understanding of individual variability and inform targeted, school‐based support.

**What's new?**

- We developed the Neurodevelopment Assessment Unit (NDAU), a child‐centred approach for assessing children referred by education practitioners for social, emotional, and/or cognitive difficulties.
- Most children showed difficulties in emotion recognition, understanding mental states, and attention. Three profiles were derived based on similarities in performance across Cognitive Systems and Social Processes domains.

**What's relevant?**

- The NDAU offers a model for dimensional assessment and tailored support aligned with children's individual profiles.
- Findings highlight variability and heterogeneity in neuropsychological profiles amongst children below diagnostic thresholds and/or with complex difficulties.

## INTRODUCTION

Mental health and neurodevelopmental conditions can have a profound and enduring impact on children and their families. However, most children with emerging mental health difficulties or neurodevelopmental features without diagnosis are often overlooked (Astle et al., 2022). Often described as the ‘missing middle’, these children may not meet criteria for specialist services, yet experience daily challenges and considerable distress (National Assembly for Wales, 2018). Research suggests that approximately 30%–40% of children display significant mental health difficulties that fall below formal diagnostic thresholds, with implications for severe mental health difficulties later in life (Copeland et al., 2015; Costello & Shugart, 1992). For children who also show signs of neurodevelopmental differences, unmet support needs may further increase vulnerability (Thapar et al., 2017). Emerging difficulties likely arise from complex, multifactorial influences, including shared genetic liability across neurodevelopmental and psychiatric conditions, as well as interactions between environmental adversity and underlying vulnerability (Riglin et al., 2020; Sameroff & Seifer, 2021; Schork et al., 2019; Thapar et al., 2017). These findings challenge the view of mental health and neurodevelopmental conditions as discrete categories, suggesting instead that many difficulties are better understood dimensionally and cut across traditional diagnostic boundaries. To better understand the strengths and needs of children with emerging difficulties, we characterised functioning using a dimensional, transdiagnostic approach and examined associations with mental health.

Given the prevalence and impact of early emerging, subthreshold difficulties, there is a clear need for greater recognition and support. The Mind Over Matter enquiry (Welsh Parliament, 2020) highlighted the urgent need for innovative, school‐based approaches to support this vulnerable group. Education professionals are well positioned to identify children who may be struggling; however, effective support requires recognition of each child's individual profile of strengths and difficulties. To address this, there is growing interest in transdiagnostic and dimensional approaches, such as the NIMH's Research Domain Criteria (RDoC) framework (Insel et al., 2010). This framework conceptualises mental health and neurodevelopment across overlapping domains of neurobehavioural functioning. Recruitment to research based on functionally defined needs (i.e., via referral from education practitioners) allows transdiagnostic approaches to capture the full range of individual variability amongst children who fall below diagnostic thresholds or have complex profiles (Astle et al., 2022; Coghill & Sonuga‐Barke, 2012).

Dimensional frameworks like RDoC are complemented by person‐centred data reduction methods, which identify subgroups of children with similar performance profiles across different transdiagnostic dimensions (Astle et al., 2022). Amongst these, the Cognitive Systems domain, which includes executive functions such as working memory, inhibition, and cognitive flexibility, has received particular attention due to its relevance for children's psychosocial and behavioural functioning (Harden et al., 2020). Research has shown that cognitive profiles can span traditional diagnostic categories (Anning et al., 2023; Astle et al., 2022) and are differentially associated with mental health outcomes (Mareva et al., 2024).

The Systems for Social Processes domain has received comparatively little attention in studies using dimensional and person‐centred approaches (Perlis, 2025). This domain broadly refers to constructs that support functioning in interpersonal contexts, including the perception and interpretation of social cues, understanding of the self, and understanding of others (Insel et al., 2010; Kozak & Cuthbert, 2016). These processes are closely linked to mental health. For example, difficulties in facial emotion recognition, a key component of perceiving and interpreting others' social cues, are associated with both psychopathology and neurodevelopmental differences (Collin et al., 2013; Crisci et al., 2025). Similarly, self‐perception is consistently linked to mental health outcomes (Sowislo & Orth, 2013). Difficulties with mentalising, or understanding others' mental states, appear to cut across both mental health and neurodevelopmental symptoms (Devine et al., 2025). Yet, to our knowledge, no studies have considered variation across both cognitive and social domains when delineating profiles of functioning in children with emerging difficulties.

Transdiagnostic and person‐centred approaches have the potential to inform tailored interventions or planned accommodations in the classroom according to functional domains underpinning children's difficulties (Cuthbert, 2022; Fletcher‐Watson, 2022; Michelini et al., 2021). In response to a lack of service frameworks for early identification and support, we developed the Neurodevelopment Assessment Unit (NDAU) as a child‐centred, school‐referral model for assessing children aged 4–7 years with social, emotional, and/or cognitive difficulties. The NDAU uses an RDoC‐informed approach and state‐of‐the‐art tools to characterise each child's strengths and provide feedback on their needs to education professionals, informing classroom‐based support and understanding. As part of this approach, the NDAU collects information from multiple informants, including both parents and teachers. A multi‐informant perspective is important because children often show different patterns of emotional and behavioural difficulties at home and at school (Goodman, 2001; Murray et al., 2021). Integrating information from both contexts provides a fuller picture of their challenges and enables targeted, context‐sensitive support (Van Goozen et al., 2022).

Here, we present an overview of our assessment approach and the first large‐scale characterisation of *N* = 486 children who were referred to the NDAU between 2017 and early 2023. Children were profiled across Cognitive Systems (e.g., language, working memory, attention) and Systems for Social Processes (e.g., social understanding, self‐perception) domains (Cuthbert, 2022; Kozak & Cuthbert, 2016). Importantly, this sample does not comprise children with pre‐existing clinical diagnoses, but rather children identified by education professionals as experiencing a variety of social, emotional, and/or cognitive difficulties in the classroom. The study was exploratory and we did not formulate hypotheses about sample characteristics or potential subgroups. Instead, we used a data‐driven approach to address the following questions: (1) What are the cognitive and social processing profiles of children referred to the NDAU? (2) Using a person‐centred latent profile approach, can distinct subgroups of children with similar patterns of cognitive and social processing performance be identified? (3) Do identified subgroups differ in behavioural and emotional difficulties as reported by parents and teachers? Prior to addressing this final question, we also examined whether profiles differed according to child (age, gender) and family characteristics (relative deprivation), as potential covariates. Together, these analyses enabled us to evaluate whether a transdiagnostic, school‐referral assessment framework can identify functionally meaningful subgroups of children that could inform tailored educational support.

## METHOD

### Ethical considerations

Ethical permission was granted by the Cardiff University School of Psychology Research Ethics Committee (ethics number: EC.16.10.11.4592GRA5). Written informed consent was obtained from each child's parent or carer, and children provided verbal assent.

### Design

A cross‐sectional approach was used to characterise individual differences in Cognitive Systems and Systems for Social Processes domains of functioning amongst children referred for classroom difficulties at a single time point. Person‐centred analytic approaches, such as latent profile or latent class analysis, are commonly applied to cross‐sectional datasets to identify subgroups of individuals who share similar patterns of functioning across multiple domains. These approaches are increasingly used in transdiagnostic research to identify meaningful profiles that capture patterns of variability in cognitive and behavioural functioning (Astle et al., 2019; Chawner et al., 2023; Poletti et al., 2018).

### Referral and participants

**FIGURE 1:**
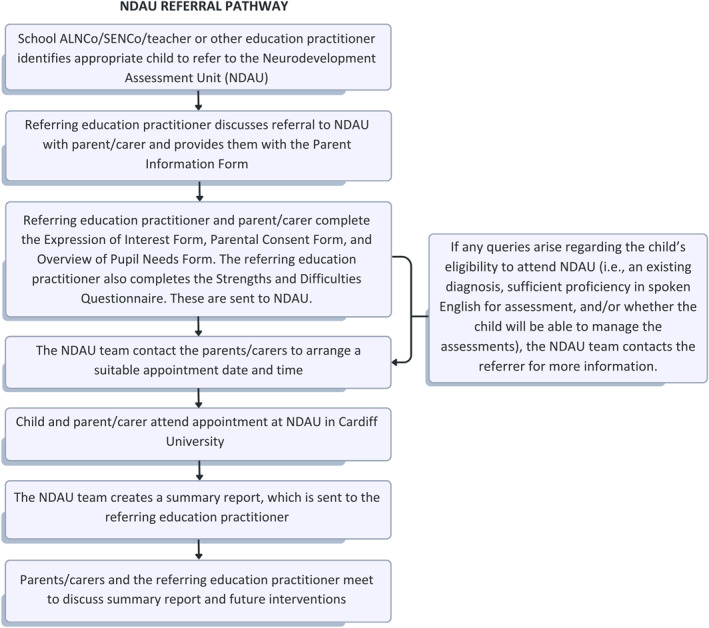
Referral pathway for Neurodevelopment Assessment Unit (NDAU). ALNCo, additional learning needs co‐ordinator; SENCo, special educational needs co‐ordinator.

The NDAU is an ongoing study at the Cardiff University Centre for Human Developmental Science (CUCHDS). The referral process is outlined in Figure 1. To date (April, 2026), over 900 children aged 4‐to‐7‐years‐old have been referred by 180 local mainstream schools. For this study, we focused on data collected from *N* = 486 children who were referred between September 2017‐January 2023 (with the unit being closed intermittently between March 2020 and March 2021 due to Covid‐19). The *M* age was 6.32 years (SD = 1.06); Three‐hundred and forty‐six children (71.2%) were male, 140 (28.8%) were female, and 387 (79.6%) were White British or White European. Only children without a diagnosis of a mental health or neurodevelopmental condition could be referred to NDAU.

Full demographic details of the sample are presented in Table 1. A substantial proportion of participating families (45.3%) had a gross household income of less than £29,999 (UK national average for 2017 was approximately £28,600 (ONS, 2017)). Nearly half of participants (48.5%) resided in the two most deprived quintiles according to the Welsh Index of Multiple Deprivation (WIMD), indicating that the sample included families residing in more deprived areas compared with the Welsh population distribution (40%).

**TABLE 1:** Sociodemographic characteristics of sample.

	*n* (%)
Child age	
4–5 years	185 (38.1%)
6–7 years	276 (56.8%)
8 years^a^	25 (5.1%)
Child sex	
Female	140 (28.8%)
Male	346 (71.2%)
Child ethnicity	
White British or White European	387 (79.6%)
European other or European British	17 (3.5%)
Pakistani, Indian, Bangladeshi, or Pakistani‐, Indian‐, Bangladeshi‐ British	11 (2.3%)
African or African British	7 (1.4%)
Caribbean or Caribbean British	5 (1.0%)
Arab or Arab British	5 (1.0%)
Other Ethnic group	3 (0.6%)
Unknown	51 (10.5%)
Family structure	
Living with birth family	420 (86.4%)
Adopted	62 (12.8%)
Looked after	4 (0.8%)
SES^b^	
WIMD quintiles, *n* (%)	
1—Most deprived (1–382)	129 (26.5%)
2 (383–764)	107 (22.0%)
3 (765–1146)	71 (14.6%)
4 (1147–1528)	52 (10.7%)
5—Least deprived (1529–1909)	108 (22.2%)
Accompanying parent or carer education^c^	
No formal qualification	57 (11.7%)
O‐levels or GCSEs	151 (31.1%)
A‐levels	95 (19.5%)
University degree	82 (16.9%)
Higher or postgraduate degree	72 (14.8%)
Household (gross) income^d^	
Up to £19,999	154 (31.7%)
£20,000–£29,999	66 (13.6%)
£30,00–£39,999	70 (14.4%)
£40,000–£49,999	49 (10.1%)
£50,000–£59,999	32 (6.6%)
£60,000+	72 (14.8%)

*Note*: Percentages are based on available data.

^a^ A small number of children were 8 years old at the time of assessment, having been referred to NDAU when they were 7 years old.

^b^ SES, socioeconomic status, based on available *n* = 467, Welsh Index of Multiple Deprivation (WIMD), WIMD ranks all small areas in Wales from 1 (most deprived) to 1909 (least deprived), quintile scores were derived from the WIMD 2019 interactive tool.

^c^ *n* = 457.

^d^ *n* = 443.

### Procedure

Children visited the NDAU with their parent or carer for two three‐hour sessions approximately a week apart. Children completed assessments with a trained researcher on computers, tablets, and using books/toys. Simultaneously, their parent or carer completed an interview and questionnaires in a separate room. Within 6 weeks of the final visit, a detailed report summarising the child's areas of strength and need, informed by their performance on a selection of assessments, was returned to the referring education practitioner. Table 2 provides an overview of selected assessments included in the feedback report pertaining to the Cognitive Systems and Systems for Social Processes domains of the RDoC framework. Individualised recommendations and suggestions of targeted evidence‐based interventions were also provided in the report by an Educational Psychologist for use at school.

**TABLE 2:** Overview of RDoC dimensions assessed in Neurodevelopment Assessment Unit in the child assessment battery included in reports returned to referring education practitioners.

Dimension	Construct/sub‐construct	Measure
Cognitive systems	Receptive language	British picture vocabulary scale (BPVS)
	Verbal reasoning	Lucid: Picture vocabulary test or link word task
	Non‐verbal reasoning	Lucid: Mental rotation task or matrix problems task
	Inhibition	NIH toolbox: Flanker
	Cognitive flexibility	NIH toolbox: Dimensional change card sort
	Verbal working memory	Automated working Memory assessment (AWMA): Backwards digit recall
	Visuospatial episodic memory	NIH toolbox: Picture sequence Memory
	Sustained attention	Amsterdam Neuropsychological tasks (ANT): Pursuit
Social processes	Facial emotion recognition	Facial emotion recognition (FER): Happy, sad, scared, angry, neutral
	Understanding mental states	False belief battery: Unexpected contents, change of location, belief‐emotion, second‐order false belief
	Self‐perception	Pictorial scale of perceived competence and social acceptance for young children (PSPCSAYC): cognitive and physical competence, peer acceptance

### Measures

#### Socioeconomic deprivation

The WIMD (Welsh Governnment, 2019) was used to estimate relative deprivation from each family's address at the time of their visit to the NDAU. The WIMD is similar to other indices of multiple deprivation but was created by the Welsh Government using indicators specific to Wales. WIMD ranks were categorised into quintiles.

#### Strengths and Difficulties Questionnaire

Parents and teachers completed the Strengths and Difficulties Questionnaire (Goodman, 1997), a widely used and well‐validated (Goodman et al., 2000) 25‐item questionnaire used to screen for emotional and behavioural difficulties in children aged 3–16 years. We profiled children based on parent‐ and teacher‐ratings of child behaviour over the last 6 months on all subscales and total difficulties.

### Cognitive Systems domain

#### Receptive language

Children's receptive vocabulary was assessed using the British Picture Vocabulary Scale (BPVS) a widely used assessment with strong evidence supporting its reliability and validity (Dunn & Dunn, 2009). In each trial, children were presented with four pictures. The researcher said one word aloud, and the child was asked to select the picture that matched with the meaning of the word. Children received four practise trials where feedback was given if incorrect. The task was terminated after children exceeded a predefined threshold of errors.

#### Verbal reasoning

Verbal reasoning was assessed using the Lucid Ability Computerised Assessment System for children aged 4–16 years, which has demonstrated good test‐retest reliability, internal consistency, and validity (GL Assessment, 2014). Four to six‐year‐olds were administered the Picture Vocabulary Test, and children over 7 years of age were given the Link Word task. Both tests terminated when the child's ability had been exceeded.

#### Non‐verbal reasoning

Non‐verbal reasoning tasks were also selected from the computer‐based Lucid Ability assessments (GL Assessment, 2014). Four‐to‐six‐year‐olds were presented with a Dressing Up mental rotation task. Children aged 7+ were given an equivalent Matrix Problems task. The tests terminated when the child's ability was exceeded.

#### Inhibitory control

Children were administered the Flanker Inhibitory Control and Attention Test from the NIH Toolbox (Toolbox Assessments, 2024), which has been validated against other neuropsychological assessments and demonstrated excellent psychometric properties (Mungas et al., 2013; Weintraub et al., 2013). Children were required to match to a target stimulus whilst inhibiting attention to its flanking stimuli presented on a computer tablet. Children's standardised scores were based on a 2‐vector scoring method that uses accuracy and reaction time in the computed score calculation.

#### Cognitive flexibility

The Dimensional Change Card Sort Test (DCCS) from the NIH Toolbox (Toolbox Assessments, 2024) was used to measure cognitive flexibility. Children were required to match bivalent test pictures to target pictures on a computer tablet. Children were presented with switch trials where, for example, after matching by shape over multiple trials, they would then be asked to match by colour for one trial, and then back to shape in the following trial. Standardised scores were based on a 2‐vector scoring method that uses accuracy and reaction time in the computed score calculation.

#### Episodic memory

The Picture Sequence Memory Test (PSMT) from the NIH Toolbox (Toolbox Assessments, 2024) was selected to assess episodic memory. Children were presented with a series of pictures on a tablet (from 6 to 18 pictures depending on age) in a sequence, and children were asked to place the images into the correct order. Children's standardised scores were based on the number of adjacent pairs placed correctly over two trials.

#### Verbal working memory

The Backward Digit Recall subtest from the Automated Working Memory Assessment (Alloway, 2007) was used to assess verbal working memory. Children were asked to recall a sequence of spoken digits in reverse. The task automatically increases in span length if there are four or more correct answers in a block, to a maximum of nine digits, and the test terminated when children made three or more incorrect responses. Correct trials were converted to standardised scores.

#### Sustained attention

The Pursuit task from the Amsterdam Neuropsychological Tasks (ANT) (De Sonneville, 1999) was used to assess sustained attention. The ANT tasks have well‐established reliability and validity (de Sonneville, 2025). Children used a mouse with their dominant hand to follow a target (a green star) that randomly moved across a computer screen for 5‐min. The mean distance (in mm) between the cursor and the moving star was calculated for each 1‐min segment of the task. Children's overall accuracy of movement was calculated as the mean distance (in mm) across the five 1‐min segments.

### Systems for Social Processes domain

#### Facial emotion recognition

Children's emotion recognition was assessed using the Facial Emotion Recognition task (FER) (Burley et al., 2022). Children were presented with 40 faces depicting happy, sad, fearful, angry, and neutral expressions from the Radboud Faces Database (Langner et al., 2010), validated for use with children (Verpaalen et al., 2019). Each facial expression was presented for 3 seconds, before the emotion category labels appeared in text on the screen, and the child was asked to identify the facial expression verbally. The presentation order of the facial expressions was pseudo‐randomised across two task versions.

#### Understanding mental states

Children's understanding of mental states was assessed using four false belief tasks which included three cognitive tasks and one affective task (Howe‐Davies et al., 2023). Children completed an adaptation of the unexpected contents task (Wellman & Liu, 2004), a version of the classic change of location false belief task (Wimmer & Perner, 1983), an adapted version of the second‐order false belief task (Perner & Wimmer, 1985), and an affective false belief task, the belief‐emotion task (Wellman & Liu, 2004). Different measures of the false belief task show good internal consistency and test‐retest reliability (Hughes et al., 2000). Children were coded as successful on each task if they provided correct responses to the test question and all control questions.

#### Child self‐perception

Children's beliefs about their own cognitive, physical, and social capabilities were assessed using the Pictorial Scale of Perceived Competence and Social Acceptance for Young Children (PSPCSAYC) (Harter & Pike, 1984), a measure that shows good internal consistency, and acceptable convergent, discriminant and predictive validity (Harter & Pike, 1984). Children completed the preschool/kindergarten version (age 4–5) or the first and second graders version (age 6–8).

### Data analysis

We report descriptive statistics (Means, SDs, and ranges) for parent and teacher ratings of children's total SDQ score and subscales, and for children's performance on assessments within the Cognitive Systems and Systems for Social Processes domains of the RDoC. For the SDQ, we used the four‐band classification system that we provide in the children's reports: ‘close to average’ (representing 80% of the population) ‘slightly raised’ (10%), ‘high’ (5%), and ‘very high’ (5%) (Youth in Mind, 2025). To aid interpretation, parent‐ and teacher‐reported SDQ total difficulties scores were compared to UK population norms (Meltzer et al., 2003). Children's performance on Cognitive Systems and Systems for Social Processes assessments was categorised using a 3‐band classification of ‘below average, average, and above average’. These categories were derived using matched population norm data for assessments in the Cognitive Systems domain (Alloway, 2007; De Sonneville, 2005; de Sonneville, 2025; Dunn & Dunn, 2009; GL Assessment, 2014; Toolbox Assessments, 2024) and community data for the Systems for Social Processes domain (Harter & Pike, 1984; Hunnikin et al., 2020; Wellman & Liu, 2004).

We next explored whether meaningful subgroups could be derived based on similarities in patterns of children's performance on the core assessments administered in NDAU. Latent profile analysis (LPA) (Gibson, 1959) is a person‐centred, data‐driven classification technique to group individuals with similar response patterns into more homogeneous subgroups, whilst maximising heterogeneity between profiles. LPA is based on mean scores and covariances on continuous variables as profile indicators. We conducted LPA using Mplus (version 8.11) using all Cognitive Systems and Systems for Social Processes assessments. We used maximum likelihood estimation with robust standard errors (MLR) as a more robust approach in the context of non‐normally distributed data and accounting for the moderate sample size (Muthén & Muthén, 2017). Starting with a single profile (*k*) model, we fitted *k*+1 models incrementally. For each model, 1000 random sets of starts were generated, and the 250 best‐fitting solutions were retained for final optimisation. Without strong theoretical rationale suggesting otherwise, variance and covariance were fixed across profiles according to Mplus defaults to reduce computational demands (Muthén & Muthén, 2018).

Model selection was based on examination of both indices of fit and subjective evaluation of models in relation to clinical interpretability (Marsh et al., 2009; Sinha et al., 2021). To aid interpretation, visualisation of profile solutions was conducted using *z*‐scores. Model fit was evaluated by sample size adjusted Bayesian Information Criterion (ssaBIC) and Bootstrapped Likelihood Ratio Tests (BLRT) (Nylund et al., 2007). Classification precision (entropy) was also examined according to recommended cut‐offs for number of latent profiles, with higher values indicating more precise classification (Wang et al., 2017). For reasons of parsimony as well as interpretability and practical utility, we avoided solutions with too many latent profiles and solutions which resulted in small profile sizes (<5% of the sample) (Nylund et al., 2007).

Finally, after identifying the latent profiles, we examined their associations with parent‐ and teacher‐reported SDQ scores using the Bolck‐Croon‐Hagenaars (BCH) approach which accounts for classification uncertainty in latent profile membership (Asparouhov & Muthén, 2014). Covariates (age, gender, and WIMD) were incorporated using the manual BCH procedure. Specifically, BCH weights were derived from the latent profile model and saved, then applied in a subsequent weighted analysis of the distal outcomes. In this step, SDQ scores were regressed on the covariates, allowing for the estimation of profile differences whilst adjusting for these variables without influencing the latent profile solution. Missing SDQ and covariate data were handled with listwise‐deletion. As all children in the sample were referred for experiencing difficulties, all profiles showed elevated levels of difficulties and therefore, selection of a ‘low risk’ group as a reference profile was not possible. For comparison purposes, we therefore selected the largest profile with relatively lower scores on the RDoC Cognitive Systems and Social Processes indicators as the reference group. Statistical significance level was defined as *p* < 0.05.

## RESULTS

### Descriptive statistics

Descriptive statistics for children's SDQ scores are presented in Table 3. Most children were rated by teachers (63.6%) and parents (70.7%) as having high to very high total difficulties on the SDQ, which compares to 7.9% and 8% in the UK general population. Parents rated higher total difficulties than teachers (*M*diff = 2.88, SE = 0.39), *t*(473) = 7.40, *p* < 0.001. Children's summary performance on assessments within the Cognitive Systems and Systems for Social Processes domains that comprised the feedback reports is available in Table 4. Within the Cognitive Systems domain, the two most prominent difficulties were sustaining attention, where 50.9% of children performed below average, and non‐verbal reasoning, where 32.1% performed below average. Notable proportions of children performed below average on the remaining assessments (18.9%–28.8%), except for verbal reasoning where 7.3% of children performed below average. Within the Systems for Social Processes domain, although performance was better for recognition of neutral and happy expressions, with 32.6% and 11.6% performing below average respectively, a substantial proportion of children showed below average performance in recognising fear (65.2%), sadness (48.6%), and anger (44.0%). As a cohort, 81.6% of children scored below average performance in recognising at least one negative emotion (i.e., sadness, fear, or anger). Over half of the sample (51.4%) performed below average on understanding mental states (theory of mind tasks).

**TABLE 3:** Profiling the sample according to parent and teacher SDQ scores.

	*M*	SD	Range	*n* (%) close to average	*n* (%) slightly raised	*n* (%) high	*n* (%) very high
Teacher SDQ							
Emotional	3.09	2.55	0–10	290 (59.7)	51 (10.5)	48 (9.9)	97 (20.0)
Conduct	3.39	2.68	0–10	216 (44.4)	48 (9.9)	51 (10.5)	171 (35.2)
Hyperactivity	7.54	2.70	0–10	104 (21.4)	87 (17.9)	56 (11.5)	239 (49.2)
Peer	3.44	2.28	0–10	182 (37.4)	123 (25.3)	82 (16.9)	99 (20.4)
Prosocial behaviour	4.42	2.90	0–10	159 (32.7)	70 (14.4)	63 (13.0)	194 (39.9)
Total problems	17.47	6.81	0–36	103 (21.2)	74 (15.2)	72 (14.8)	237 (48.8)
Parent SDQ							
Emotional	4.13	2.68	0–10	212 (44.7)	67 (14.1)	97 (20.5)	98 (20.7)
Conduct	4.67	2.68	0–10	111 (23.4)	62 (13.1)	126 (26.6)	175 (36.9)
Hyperactivity	8.03	2.40	0–10	75 (15.8)	71 (15.0)	62 (13.1)	266 (56.1)
Peer	3.51	2.33	0–10	171 (36.1)	81 (17.1)	66 (13.9)	156 (32.9)
Prosocial behaviour	6.31	2.58	0–10	177 (37.3)	74 (15.6)	59 (12.4)	164 (34.6)
Total problems	20.32	6.87	2–37	92 (19.4)	47 (9.9)	62 (13.1)	273 (57.6)

*Note*: Teacher SDQ *N* = 486; Parent SDQ *n =* 474; Categories were based on the four‐band classification system (Youth in Mind, 2025); For prosocial behaviour, scores indicate close to average, slightly lowered, low, and very low levels of prosocial behaviour.

Abbreviation: SDQ, Strengths and Difficulties Questionnaire.

**TABLE 4:** Descriptive statistics for dimensional constructs/sub‐constructs for total sample.

	*N*	*M*	SD	Range	*n* (%) below average	*n* (%) average	*n* (%) above average
Cognitive systems							
Receptive language^1^	470	93.87	13.07	70–138	113 (24.0%)	332 (70.6%)	25 (5.3%)
Verbal reasoning^1^	452	104.56	14.61	60–151	33 (7.3%)	323 (71.5%)	96 (21.2%)
Non‐verbal reasoning^1^	402	94.59	18.12	60–150	**129 (32.1%)**	225 (56.0%)	48 (11.9%)
Inhibition^1^	430	90.48	14.04	54–131	124 (28.8%)	299 (69.5%)	7 (1.6%)
Cognitive flexibility^1^	439	92.83	14.34	41–149	123 (28.0%)	303 (69.0%)	13 (3.0%)
Verbal working memory^1^	397	97.92	16.23	64–141	75 (18.9%)	274 (69.0%)	48 (12.1%)
Visuospatial episodic memory^1^	447	98.47	19.47	51–146	97 (21.7%)	258 (57.7%)	92 (20.6%)
Sustained attention^2^	407	78.50	27.46	40–124.30	**207 (50.9%)**	180 (44.2%)	20 (4.9%)
Social processes							
Emotion recognition; happy^3^	457	88.98	17.14	0–100	53 (11.6%)	146 (31.9%)	258 (56.5%)
Emotion recognition; sad^3^	457	67.62	21.28	0–100	**222 (48.6%)**	106 (23.2%)	129 (28.2%)
Emotion recognition; fear^3^	457	53.69	28.05	0–100	**298 (65.2%)**	74 (16.2%)	85 (18.6%)
Emotion recognition; angry^3^	457	67.29	27.86	0–100	**201 (44.0%)**	82 (17.9%)	174 (38.1%)
Emotion recognition; neutral^3^	457	73.93	28.59	0–100	**149 (32.6%)**	57 (12.5%)	251 (54.9%)
Understanding mental states^4^	469	2.09	1.33	0–4	**241 (51.4%)**	179 (38.2%)	49 (10.4%)
Self‐perception; cognitive^5^	439	2.99	0.66	1–4	40 (9.1%)	190 (43.3%)	209 (47.6%)
Self‐perception; physical^5^	437	2.88	0.63	1.17–4	47 (10.8%)	216 (49.4%)	174 (39.8%)
Self‐perception; peer^5^	436	2.88	0.65	1–4	52 (11.9%)	213 (48.9%)	171 (39.2%)

*Note*: Percentages were calculated based on available data for each assessment. Assessments where >30% of children scored below average are bold. Categories were based on: ^1^Age‐corrected standard scores (*M* = 100, SD = 15). Scores of 85–115 indicated average performance for age; ^2^Raw scores converted to age‐normed *z*‐scores (de Sonneville, 2025), reversed, scores <–4 were set to −4 to limit disproportionate influence of outliers, then standardised (*M* = 100, SD = 15). Scores of 85–115 represented average performance; ^3^Data from a comparison sample (Hunnikin et al., 2020, 2022), where scores of 66.67%–80.00% indicated the average range (except for happy which ranged from 66.67% to 90.00%); ^4^Expected performance for the child's age: 4–6 years passing 2–3 tasks indicated average performance, and 7 years and above, passing all tasks indicated average performance. ^5^Scores of 2.1–3 (responses of slightly below to slightly above average) for cognitive and physical competence, and peer acceptance indicated the average range.

### Neurodevelopmental profiles

**FIGURE 2:**
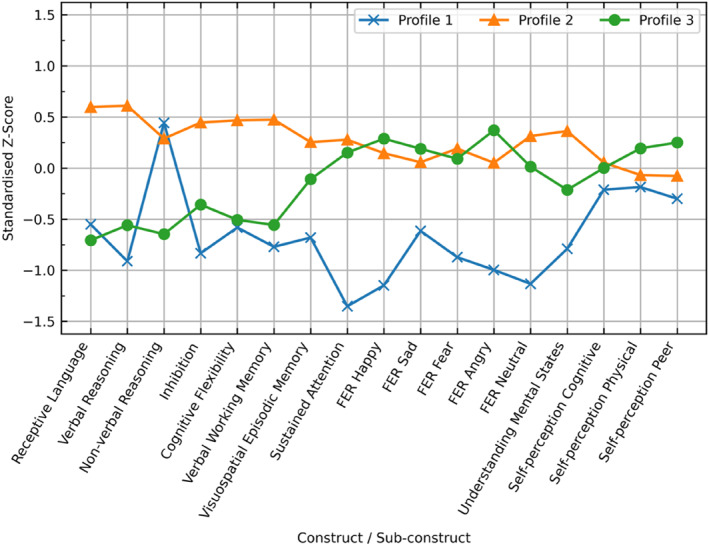
Visualisation of latent profile analysis 3‐profile solution for all constructs/sub‐constructs (*z*‐scores) in the Cognitive Systems and Social Processes domains. Profile 1 (*n* = 78, 16%): Pronounced, Pervasive Social Processes and Cognitive Systems Difficulties, Profile 2 (*n* = 251, 51.6%): Moderate Social Processes Difficulties, Profile 3 (*n* = 157, 32.3%): Moderate Social Processes and Cognitive Systems Difficulties. FER; facial emotion recognition.

We examined profile solutions ranging from one to four profiles, with fit statistics for each model presented in Table 5. Across models, the sample‐size adjusted Bayesian Information Criterion (ssaBIC) continued to decrease, suggesting improved model fit with a greater number of profiles. Bootstrapped Likelihood Ratio Tests were also significant for all models (*p*s < 0.001), indicating that a greater number of profiles improved model fit. Entropy for the three‐ and four‐class solutions exceeded 0.76, associated with 90% correct assignment. However, the smallest profile in the four‐profile solution comprised less than 5% of the sample (19 children), raising concerns about the stability, interpretability, and clinical utility of the subgroup. Furthermore, visual inspection of the four‐profile solution (see Supporting Information S1: Figure S1) showed that the profiles were largely similar in shape, whereas the three‐profile solution showed clearer differentiation in performance across Cognitive Systems and Systems for Social Processes domains (see Figure 2). In line with recommendations that latent profiles should differ in profile shape rather than overall elevation (Marsh et al., 2009), the three‐profile solution was judged to offer more meaningful distinctions. For these reasons, we selected the three‐profile solution as the most parsimonious and interpretable model.

**TABLE 5:** Statistical indices of fit for *k* + 1 latent profile analysis models (*N* = 486).

N Latent profiles	Free parameters	Log‐likelihood	ssaBIC	Entropy	Minimum profile size^a^	BLRT
1	34	−27260.378	54,623.172		486 (100%)	
2	52	−26968.823	54,094.283	0.772	165 (33%)	*p* < 0.001
3^b^	70	−26824.024	53,858.906	0.783	78 (16%)	*p* < 0.001
4	88	−26734.346	53,733.772	0.813	19 (4%)	*p* < 0.001

Abbreviations: BLRT, bootstrapped likelihood ratio tests; ssaBIC, Bayesian information criterion sample size adjusted.

^a^ According to most likely profile membership.

^b^ Final model selected.

Children's performance across assessments according to profile membership is available in Table 6, and categorical descriptive statistics using the 3‐band classification of ‘below average, average, and above average’ are presented in Supporting Information S1: Table S1. Visualisation of children's performance using *z*‐scores is presented in Figure 2. There were notable proportions of children (i.e., >30%) in each subgroup who had difficulty recognising negative emotions. Profile 1: Pronounced, Pervasive Social Processes and Cognitive Difficulties (*n* = 78, 16%) showed difficulties across all assessments except verbal and non‐verbal reasoning and self‐perception and considerable difficulties with emotion recognition and attention. Many children in Profile 2: Moderate Social Processes Difficulties (*n* = 251, 51.6%) showed average or above average performance across the Cognitive Systems domain, yet a notable proportion showed difficulties in sustained attention. Many children in this group showed below average performance on recognising negative emotions and understanding mental states. Finally, Profile 3: Moderate Social Processes and Cognitive Difficulties (*n* = 157, 32.3%) showed difficulties across cognitive assessments, but not as pronounced as Profile 1. They also showed difficulty in non‐verbal reasoning and moderate difficulties recognising negative emotions and understanding mental states.

**TABLE 6:** Descriptive statistics for children's performance on cognitive systems and social processes dimensions and SDQ scores by profile.

	Profile 1: Pronounced, pervasive social processes and cognitive systems difficulties (*n* = 78)	Profile 2: Moderate social processes difficulties (*n* = 251)	Profile 3: Moderate social processes and cognitive difficulties (*n* = 157)
Cognitive systems			
Receptive language	86.67 (10.12)	101.68 (11.10)	84.64 (8.50)
Verbal reasoning	91.23 (15.41)	113.46 (10.42)	96.39 (9.71)
Non‐verbal reasoning	102.59 (14.97)	99.84 (17.39)	82.12 (13.84)
Inhibition	78.78 (13.87)	96.74 (12.38)	85.45 (13.84)
Cognitive flexibility	84.48 (12.94)	99.54 (12.24)	85.56 (12.61)
Verbal working memory	85.41 (15.66)	105.61 (13.53)	88.88 (13.31)
Visuospatial episodic memory	85.23 (15.52)	103.41 (19.01)	96.34 (18.76)
Sustained attention	55.10 (21.21)	86.46 (25.72)	76.34 (26.37)
Social processes			
Emotion recognition; happy	69.30 (25.87)	91.48 (12.91)	93.92 (11.00)
Emotion recognition; sad	54.53 (26.46)	68.83 (19.22)	71.64 (19.61)
Emotion recognition; fear	29.23 (28.46)	59.05 (25.23)	56.25 (26.50)
Emotion recognition; angry	39.52 (26.59)	68.72 (25.87)	77.58 (22.96)
Emotion recognition; neutral	41.54 (32.86)	82.90 (19.81)	74.33 (27.96)
Understanding mental states	1.04 (1.03)	2.57 (1.22)	1.81 (1.27)
Self‐perception cognitive	2.86 (0.65)	3.03 (0.69)	3.00 (0.59)
Self‐perception physical	2.76 (0.60)	2.83 (0.62)	3.00 (0.64)
Self‐perception peer	2.69 (0.60)	2.83 (0.65)	3.05 (0.64)

*Note*: Mean (SD).

Abbreviation: SDQ, Strengths and Difficulties Questionnaire.

### Neurodevelopmental profile differences according to sociodemographic factors and child strengths and difficulties

There was no evidence of differences in profile membership according to child sex, *χ* ^2^(2, 486) = 0.52, *p* = 0.77 (74.4% male in Profile 1, 70.1% in Profile 2, 71.3% in Profile 3). However, sex was retained as covariate given the higher proportion of males in the sample. Groups differed according to child age *F*(2, 483) = 40.85, *p* < 0.001, where children in Profile 1 (*M*age [months] = 66.13, SE = 1.27) were significantly younger than those in Profiles 2 (*M*age [months] = 75.71, SE = 0.78, *p* < 0.001) and 3 (*M*age [months] = 80.92, SE = 0.90, *p* < 0.001) and children in Profile 3 were significantly older than those in Profile 2, *p* < 0.001. The groups also differed according to socioeconomic deprivation, as estimated by WIMD quintiles, *F*(2,464) = 5.19, *p* = 0.006, where children in Profile 1 (*M* = 2.40, SE = 0.17) lived in more deprived areas than children in Profile 2 (*M* = 2.99, SE = 0.10, *p* = 0.01), with no differences between Profile 3 (*M* = 2.66, SE = 0.12).

With models adjusted for child sex, age, and estimated socioeconomic deprivation (WIMD), we identified a significant difference between profiles according to teacher total rated difficulties on the SDQ, where children in Profile 3 had significantly higher total difficulties than children in Profile 2. Children in Profiles 1 and 3 had significantly higher teacher‐rated hyperactivity difficulties than children in Profile 2. No significant differences were detected between profiles according to parent‐rated total difficulties. Children in Profile 1 had lower parent‐rated emotional difficulties than children in Profile 2. Children in Profile 3 had lower parent‐reported peer problems than children in Profile 2, see Table 7.

**TABLE 7:** Means and SEs of parent‐ and teacher‐reported SDQ total and subscale difficulties by profile.

	Profile 2 (ref) Mean (SE)	Profile 1 Mean (SE)	Wald *χ* ^2^	*p*‐value	Profile 3 Mean (SE)	Wald *χ* ^2^	*p*‐value
Teacher reports							
Emotional difficulties	2.90 (0.18)	2.90 (0.32)	0.00	0.99	3.43 (0.26)	2.31	0.13
Peer difficulties	3.34 (0.16)	3.94 (0.32)	3.02	0.08	3.47 (0.22)	0.19	0.67
Conduct difficulties	3.33 (0.19)	3.54 (0.35)	0.27	0.61	3.54 (0.28)	0.30	0.58
Hyperactivity difficulties	**7.16 (0.20)**	**8.02 (0.32)**	**5.50**	**0.02**	**8.13 (0.24)**	**8.23**	**0.004**
Prosocial behaviour	4.72 (0.19)	4.02 (0.38)	2.78	0.10	3.98 (0.29)	3.69	0.06
Total difficulties	**16.73 (0.51)**	18.47 (0.83)	3.40	0.07	**18.57 (0.66)**	**4.01**	**0.045**
Parent reports							
Emotional difficulties	**4.53 (0.21)**	**3.69 (0.30)**	**5.68**	**0.02**	3.82 (0.27)	3.65	0.06
Peer difficulties	**3.84 (0.17)**	3.75 (0.32)	0.07	0.80	**3.16 (0.23)**	**4.70**	**0.03**
Conduct difficulties	4.57 (0.19)	5.36 (0.38)	3.45	0.06	4.76 (0.30)	0.22	0.64
Hyperactivity difficulties	8.12 (0.17)	8.13 (0.32)	0.00	0.98	8.18 (0.25)	0.03	0.87
Prosocial behaviour	6.29 (0.19)	6.31 (0.35)	0.00	0.96	6.14 (0.28)	0.18	0.67
Total difficulties	21.01 (0.50)	20.91 (0.86)	0.01	0.92	19.94 (0.74)	1.19	0.28

*Note*: Analyses controlled for sex, age, and relative deprivation (Welsh Index of Multiple Deprivation scores; WIMD); Analyses were based on *N* = 466 with available SDQ, age, sex, and WIMD data. Comparisons were made against Profile 2 (ref). Significant differences (*p* < 0.05) are indicated in bold.

Abbreviations: SE, standard error; SDQ, Strengths and Difficulties Questionnaire.

## DISCUSSION

Research‐driven dimensional assessment of young children referred by education practitioners contributes to understanding the heterogenous and complex profiles of children who experience difficulties in the classroom. In addition to scientific enquiry, we present the Neurodevelopment Assessment Unit (NDAU) as a model of service delivery offering a much‐needed, free, and timely service to parents, children, and practitioners. This model provides practitioners with an accurate picture of a child's individual needs and supports selection of appropriate interventions (Van Goozen et al., 2022).

### A dimensional approach to the assessment of young, school‐referred children

Our key aim was to provide an overview of the first cohort of children referred to the NDAU. The most pronounced difficulties were observed in sustaining attention, recognising negative emotions, and understanding mental states, with almost half of children performing below average on these assessments. Although prior transdiagnostic research has highlighted the cognitive needs of children who are struggling in school (Astle et al., 2019), our findings emphasise the critical importance of addressing socio‐emotional function in these children. This is particularly important given that the Systems for Social Processes domain has been comparatively underexplored in dimensional and person‐centred research, despite its clear relevance for mental health outcomes (Perlis, 2025). Many constructs within this domain can be targeted with relatively straightforward ‘low level’ interventions, such as age‐appropriate activities that support the development of specific social‐cognitive skills, such as emotion recognition training (Hunnikin et al., 2020, 2022; Wells et al., 2021) and interventions that support children's developing understanding of mental states, such as story book reading and sociodramatic play (Hofmann et al., 2016). Taken together, this comprehensive dimensional approach to assessment elucidates specific areas where many children may benefit from additional monitoring, support, or adjustments in the classroom environment (Astle et al., 2022).

### Transdiagnostic profiles

A secondary aim was to use person‐centred methods to identify subgroups of children who shared similar cognitive and socio‐emotional profiles. We found three distinct subgroups. The Pronounced, Pervasive Social and Cognitive Difficulties group (Profile 1: 16% of the sample) was characterised by below average scores across almost all assessments, and marked difficulties with sustained attention, emotion recognition and understanding mental states.

The Moderate Social Processes Difficulties group included the largest proportion of individuals (Profile 2: 51.6%). Although this profile showed scores close to the sample mean on several measures, it is important to note that the latent profiles were derived from a sample of children all referred for classroom difficulties. As such, scores near zero reflect functioning relative to this referred sample, rather than the general population. When considered against population norms, children in this profile still show areas of difficulty in attention as well as in the recognition of negative emotions and understanding mental states. Overall, this group presented with a less distinct pattern of difficulties compared to the other two profiles but nonetheless demonstrated meaningful difficulties in Social Processes and Cognitive Systems domains and had higher parent‐reported emotional and peer difficulties than the other two profiles. One possible explanation for this pattern is that, despite relatively less pronounced difficulties in the assessed cognitive and social processes domains, children in this profile may experience challenges in other RDoC domains. Positive and Negative Valence Systems, for example, encompass processes related to reward and motivation, as well as responses to threat, loss, and frustrative non‐reward (Insel et al., 2010; Kozak & Cuthbert, 2016), and have been consistently linked to multiple forms of psychopathology, particularly anxiety and depression (Proudfit et al., 2015). These domains were not captured in the current analyses but may contribute to elevated emotional and peer‐related difficulties in this profile.

Finally, the Moderate Social Processes and Cognitive Difficulties group (Profile 3: 32.3% of the sample) was characterised by difficulties across Cognitive Systems and Social Processes, yet their socio‐emotional difficulties these were less substantial than the Pronounced, Pervasive Difficulties group. Notable proportions of children in this group had difficulties across cognitive tasks and with recognising emotions and understanding mental states. Interestingly, children within these groups did not differ significantly based on parent behavioural reports, but the groups did differ by teacher SDQ reports. Specifically, children in Profiles 1 and 3 had higher teacher‐reported hyperactivity problems, likely reflecting the greater support needs these subgroups have in certain cognitive domains. This divergence between informants highlights the value of a multi‐informant approach, as children's difficulties may manifest differently across home and school contexts (Goodman, 2001; Murray et al., 2021), and relying on a single informant may obscure meaningful variation between subgroups.

The presence of these three profiles provides new insight into how difficulties in the cognitive and socio‐emotional domains may co‐occur in groups of children who are identified as having difficulties at school. This dimensional transdiagnostic approach allows for characterisation of the overlapping constructs that may underpin emerging mental health difficulties and neurodevelopmental differences (Astle et al., 2022; Michelini et al., 2021). A key strength of this approach is the inclusion of constructs across two domains of RDoC, including Systems for Social Processes, which complements and extends prior research that has focused on cognitive profiles (Astle et al., 2019; Bathelt et al., 2018). In doing so, the present study addresses a notable gap in the literature, as previous person‐centred studies have rarely incorporated variation across both cognitive and social domains when delineating profiles of functioning. Further research with NDAU data, which includes assessments across additional RDoC domains, such as Positive and Negative Valance Systems, Arousal Regulatory systems, and Sensorimotor systems (Cuthbert, 2022; Insel et al., 2010) can provide more comprehensive profiles and may shed light on whether the larger profile could be broken down into more targeted profiles of function (Cuthbert, 2022; Insel et al., 2010).

The exploratory investigation of subgroups represents a preliminary step that requires further replication and integration with findings from other samples and assessment tools. Nonetheless, our findings underscore that using profile analysis in conjunction with a transdiagnostic approach can identify subgroups of children who can be distinguished by subtle differences in performance (Astle et al., 2022; Michelini et al., 2021). With careful implementation, this approach has great potential as a framework for school staff to understand common profiles of cognitive and social processing difficulties amongst children who struggle in school and facilitate effective support. It is, however, essential to acknowledge that individual variability remains within each subgroup. As such, whilst this framework supports more nuanced understanding and formulation of support, personalised assessment and intervention remain crucial.

### Limitations

The current results must be interpreted within the context of some limitations. First, females are underrepresented in the NDAU sample. This may be the result of health and education professionals more readily identifying boys than girls as displaying difficulties (e.g., learning difficulties and wider cognitive difficulties) (Guy et al., 2022). However, a higher proportion of boys is also consistent with prevalence estimates reported in cohort studies of various developmental disorders (Russell et al., 2014) and in referred samples (Astle et al., 2019). Additionally, although representative of the local population, the sample was still predominantly White British or White European (79.3%), limiting generalisability to more ethnically diverse populations. It is also important to note the limitations of LPA. Our chosen 3‐profile solution was most reasonable based on distinctiveness of profiles, interpretability, and clinical utility (Sinha et al., 2021). However, the more objective approach of determining the best solution statistically was of limited use, given that additional profiles improved model fit but yielded very small and less meaningful subgroups. There are no definitive rules for selecting the ‘right’ number of profiles, and the optimal solution may vary depending on sample size (Marsh et al., 2009). Replication using person‐centred approaches with larger samples of school‐referred children experiencing difficulties is required.

## CONCLUSIONS

Our findings highlight the heterogeneity of children's profiles of cognitive and socio‐emotional development, as well as first indications of potential subgroups who share similar patterns of performance across these domains. By integrating both Cognitive Systems and the comparatively underrepresented Systems for Social Processes domain, alongside information from multiple informants, this study provides a more comprehensive account of children's functioning across contexts. Although replication is necessary, the integration of dimensional assessment into educational practise may have the potential to inform individualised support, resource allocation, and create classroom environments where children can flourish.

## AUTHOR CONTRIBUTIONS

**Amy L. Paine**: Conceptualization; data curation; formal analysis; investigation; methodology; supervision; validation; visualization; writing—original draft preparation; writing—review and editing. **Steve Eaton**: Conceptualization; data curation; investigation; project administration; resources; software; supervision; writing—review and editing. **Aikaterini Bekiropoulou**: Formal analysis; validation; visualization; writing—review and editing. **Christopher Hobson**: Conceptualization; Investigation; methodology; project administration; supervision; writing—review and editing. **Cerith Waters**: Conceptualization; funding acquisition; investigation; methodology. **Anita Thapar**: Conceptualization; funding acquisition; investigation; methodology; writing—review and editing. **Katherine H. Shelton**: Conceptualization; Investigation; methodology; writing—review and editing. **Kate Langley**: Conceptualization; investigation; methodology; writing—review and editing. **Catherine R. G. Jones**: Conceptualization; investigation; methodology; writing—review and editing. **Dale F. Hay**: Conceptualization; funding acquisition; investigation; methodology; writing—review and editing. **Susan R. Leekam**: Conceptualization; funding acquisition; investigation; methodology; writing—review and editing. **Elisabeth von dem Hagen**: Conceptualization; investigation; methodology; writing—review and editing. **The Neurodevelopment Assessment Unit Team**: Data curation; investigation. **Stephanie H. M. van Goozen**: Conceptualization; investigation; methodology; project administration; resources; software; supervision; writing—review and editing.

## CONFLICT OF INTEREST STATEMENT

The authors declare no conflicts of interest.

## ETHICAL CONSIDERATIONS

Ethical permission was granted by the Cardiff University School of Psychology Research Ethics Committee (ethics number: EC.16.10.11.4592GRA5) on 8/11/2016. Written informed consent was obtained from each child's parent or carer, and teachers and children provided verbal assent.

## Supporting information

Supporting Information S1

## Data Availability

Data from NDAU used in this study is managed by the research team and can be made available to researchers following approval from the Cardiff University School of Psychology Research Ethics Committee and the data owners, compliance with the EU General Data Protection Regulation (GDPR), and with relevant collaboration agreements in place. The consent given by the participants does not permit for storage of data in repositories or journals. Researchers who would like access to data sets should email ndau@cardiff.ac.uk.
