# Supplementary material for: A new approach to assessment for young children referred by education professionals for socio‐emotional, behavioural, and cognitive difficulties
Source: JCPP Adv. 2026 Jun 3:e70138. Online ahead of print. doi: 10.1002/jcv2.70138 (PMC13339069; doi:10.1002/jcv2.70138)
Supplement: Supplementary file 1 — Supporting Information S1 [file JCV2-9999-e70138-s001.docx]

**A new approach to assessment for young children referred by education professionals for socio-emotional, behavioural, and cognitive difficulties**

**Supporting Information**

**Table S1.** Categories of children’s performance on Cognitive Systems and Social Processes dimensions by profile.

|  |  | Profile 1: Pronounced, Pervasive Social Processes and Cognitive Difficulties  (*n* = 78) | | | Profile 2: Moderate Social Processes Difficulties (*n* = 251) | | | Profile 3: Moderate Social Processes and Cognitive Difficulties (*n* = 157) | | |
| --- | --- | --- | --- | --- | --- | --- | --- | --- | --- | --- |
|  |  | *n* (%) Below average | *n* (%) Average | *n* (%) Above average | *n* (%) Below average | *n* (%) Average | *n* (%) Above average | *n* (%) Below average | *n* (%) Average | *n* (%) Above average |
| Cognitive Systems | Receptive language^1^ | **28 (38.9)** | 44 (61.1) | 0 (0.0) | 10 (4.1) | 211 (85.8) | 25 (10.2) | **75 (49.3)** | 77 (50.7) | 0 (0.0) |
|  | Verbal reasoning^1^ | 19 (27.5) | 47 (68.1) | 3 (4.3) | 1 (0.4) | 145 (61.2) | 91 (38.4) | 13 (8.9) | 131 (89.7) | 2 (1.4) |
|  | Non-verbal reasoning^1^ | 7 (12.1) | 41 (70.7) | 10 (17.2) | 49 (22.7) | 129 (59.7) | 38 (17.6) | **73 (57.0)** | 55 (43.0) | 0 (0.0) |
|  | Inhibition^1^ | **35 (58.3)** | 25 (41.7) | 0 (0.0) | 31 (13.7) | 189 (83.3) | 7 (3.1) | **58 (40.6)** | 85 (59.4) | 0 (0.0) |
|  | Cognitive flexibility^1^ | **34 (54.8)** | 28 (45.2) | 0 (0.0) | 27 (11.6) | 193 (82.8) | 13 (5.6) | **62 (43.1)** | 82 (56.9) | 0.0 |
|  | Verbal working memory^1^ | **22 (53.7)** | 18 (43.9) | 1 (2.4) | 12 (5.4) | 167 (74.9) | 44 (19.7) | **41 (30.8)** | 89 (66.9) | 3 (2.3) |
|  | Visuospatial episodic memory^1^ | **30 (46.9)** | 30 (46.9) | 4 (6.3) | 32 (13.6) | 141 (60.0) | 62 (26.4) | 35 (23.6) | 87 (58.8) | 26 (17.6) |
|  | Sustained attention^2^ | **53 (89.8)** | 5 (8.5) | 1 (1.7) | **84 (39.3)** | 113 (52.8) | 17 (7.9) | **70 (52.2)** | 62 (46.3) | 2 (1.5) |
| Social Processes | Emotion recognition; happy^3^ | **27 (39.7)** | 31 (45.6) | 10 (14.7) | 18 (7.5) | 79 (33.1) | 142 (59.4) | 8 (5.3) | 36 (24.0) | 106 (70.7) |
|  | Emotion recognition; sad^3^ | **48 (70.6)** | 10 (14.7) | 10 (14.7) | **110 (46.0)** | 59 (24.7) | 70 (29.3) | **64 (42.7)** | 37 (24.7) | 49 (32.7) |
|  | Emotion recognition; fear^3^ | **60 (88.2)** | 3 (4.4) | 5 (7.4) | **147 (61.5)** | 41 (17.2) | 51 (21.3) | **91 (60.7)** | 30 (20.0) | 29 (19.3) |
|  | Emotion recognition; angry^3^ | **59 (86.8)** | 3 (4.4) | 6 (8.8) | **101 (42.3)** | 50 (20.9) | 88 (36.8) | 41 (27.3) | 29 (19.3) | 80 (53.3) |
|  | Emotion recognition; neutral^3^ | **51 (75.0)** | 7 (10.3) | 10 (14.7) | 51 (21.3) | 33 (13.8) | 155 (64.9) | **47 (31.3)** | 17 (11.3) | 86 (57.3) |
|  | Understanding mental states^4^ | **53 (73.6)** | 19 (26.4) | 0 (0.0) | **82 (33.3)** | 122 (49.6) | 42 (17.1) | **106 (70.2)** | 38 (25.2) | 7 (4.6) |
|  | Self-perception; cognitive^5^ | 6 (10.0) | 30 (50.0) | 24 (40.0) | 24 (10.1) | 93 (39.2) | 120 (50.6) | 10 (7.0) | 67 (47.2) | 65 (45.8) |
|  | Self-perception; physical^5^ | 8 (13.3) | 34 (56.7) | 18 (30.0) | 26 (11.0) | 119 (50.4) | 91 (38.6) | 13 (9.2) | 63 (44.7) | 65 (46.1) |
|  | Self-perception; peer^5^ | 11 (19.0) | 34 (58.6) | 13 (22.4) | 31 (13.1) | 117 (49.6) | 88 (37.3) | 10 (7.0) | 62 (43.7) | 70 (49.3) |

*Note.* *Note.* Percentages were calculated based on available data for each assessment. Assessments where > 30% of children scored below average are bold. Categories were based on: ^1^Age-corrected standard scores (*M* = 100, *SD* = 15). Scores of 85–115 indicated average performance for age; ^2^Raw scores converted to age-normed *z*-scores (de Sonneville, 2025), reversed, scores <–4 were set to –4 to limit disproportionate influence of outliers, then standardized (*M* = 100, *SD* = 15). Scores of 85–115 represented average performance; ^3^Data from a comparison sample (Hunnikin et al., 2020, 2022), where scores of 66.67 to 80.00% indicated the average range (except for happy which ranged from 66.67 to 90.00%); ^4^Expected performance for the child’s age: 4-6 years passing 2-3 tasks indicated average performance, and 7 years and above, passing all tasks indicated average performance. ^5^Scores of 2.1-3 (responses of slightly below to slightly above average) for cognitive and physical competence, and peer acceptance indicated the average range.


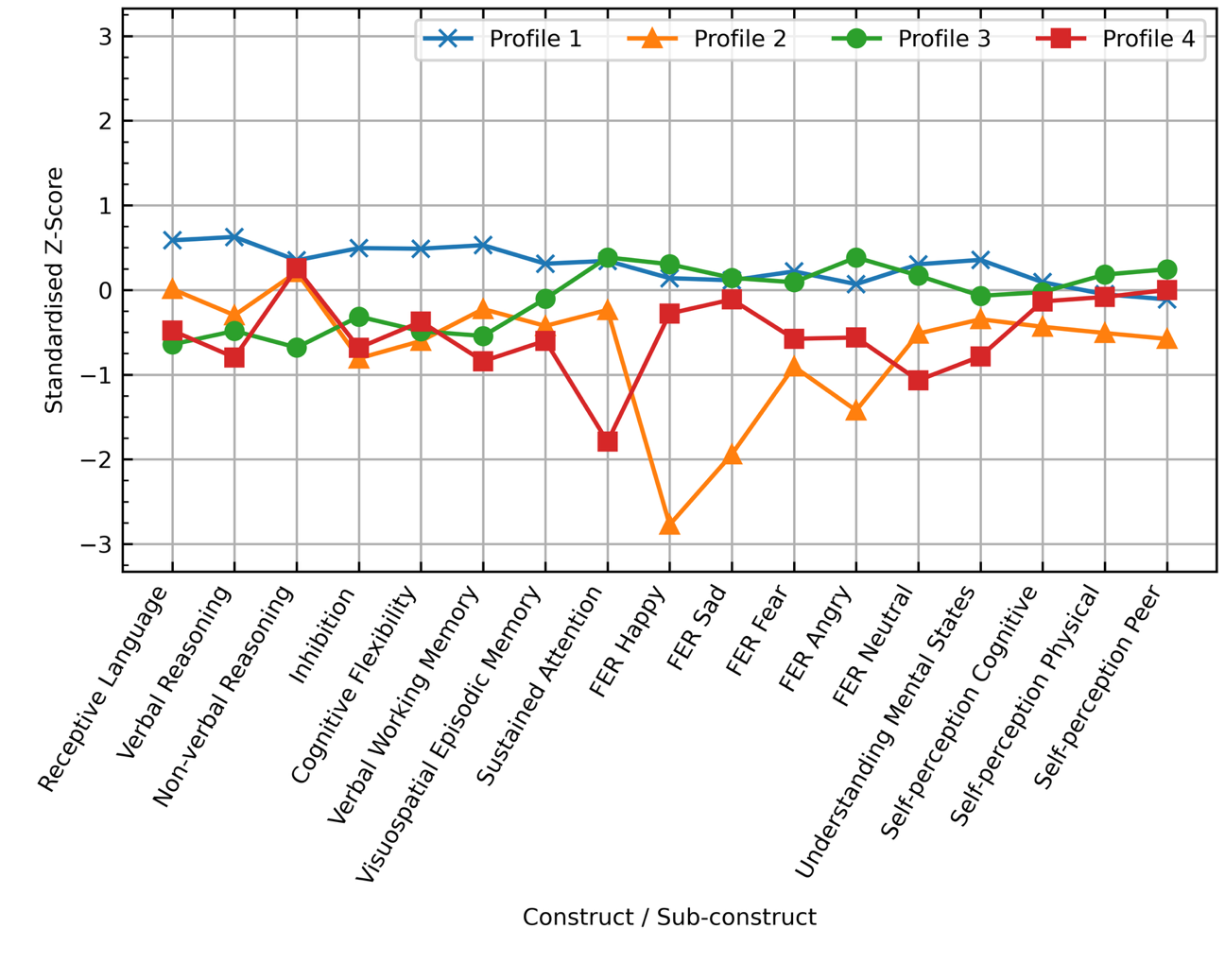


**Figure S1.** Visualisation of 4-class Solution Latent Profile Analysis for all Constructs/Sub-constructs (*z*-scores) in the Cognitive Systems and Social Processes Domains. FER: Facial Emotion Recognition. For an overview of measures, see Table 1 of the main manuscript.
